# Decomposition of multivariate function using the Heaviside step function

**DOI:** 10.1186/2193-1801-3-704

**Published:** 2014-11-29

**Authors:** Eisuke Chikayama

**Affiliations:** Department of Information Systems, Niigata University of International and Information Studies, 3-1-1 Mizukino, Nishi-ku, Niigata-shi, Niigata, 950-2292 Japan; Environmental Metabolic Analysis Research Team, RIKEN, 1-7-22 Suehiro-cho, Tsurumi-ku, Yokohama-shi, Kanagawa, 230-0045 Japan; Image Processing Research Team, RIKEN, 2-1 Hirosawa, Wako-shi, Saitama, 351-0198 Japan

**Keywords:** Heaviside step function, Dirac delta function, Transform

## Abstract

Whereas the Dirac delta function introduced by P. A. M. Dirac in 1930 to develop his theory of quantum mechanics has been well studied, a not famous formula related to the delta function using the Heaviside step function in a single-variable form, also given by Dirac, has been poorly studied. Following Dirac’s method, we demonstrate the decomposition of a multivariate function into a sum of integrals in which each integrand is composed of a derivative of the function and a direct product of Heaviside step functions. It is an extension of Dirac’s single-variable form to that for multiple variables.

## 1. Introduction

P.A. M. Dirac introduced in 1930 a function, now called the Dirac delta function, to develop his theory of quantum mechanics (Dirac 1958). It takes value zero at *x* ≠ 0 and its integral is unity. A fundamental property derivable from the definition of the Dirac delta function is that any multivariate real function can be expressed with delta functions δ and integrals as follows, 1.1

The importance of this property is analogous to the Fourier transform (Bracewell 1965) for its ability to yield an alternative representation of any multivariate function in which the variables of the function are changed. The delta function can be seen in applications from physics to engineering: such as quantum mechanical states (Lee 1992); quantum similarity integrals (Safouhi and Berlu 2006); pseudopotential (Derevianko 2003); a spin system with a classical environment (Calvani et al. 2013); and generally, numbers of formulae in the Fourier and Laplace transforms, and differential equations (Schwartz 1966; Kreyszig 2011). A more rigorous mathematical theory for the delta function has also been developed and expanded under the branch in pure mathematics called the theory of distributions by L. Schwartz (Schwartz 1945). Further development is the generalized delta impulse (Corinthios 2003), which is an extension of the Dirac delta function to that on the complex plane and is applied to theories of generalized Laplace, *z*, Hilbert, and Fourier-related transforms (Corinthios 2005, 2007).

Transforming the integral expression in one variable using the Dirac delta function δ into one using the Heaviside step function σ,
1.2

is essentially described in Dirac’s quantum mechanics text (Dirac 1958). It is derived from the relation between the Dirac delta function and the derivative of the Heaviside step function. As these two expressions, the left- and right-hand sides of (1.2), are mathematically equivalent, the step-function expression would be expected to find potent applications in physics and engineering. One general example must be approximation theory (Milovanovic and Rassias 2014). The other example is replacement of the Dirac delta functions with the Heaviside step functions by which (divergent) delta functions can be hidden from integrand. It helps a rigorous formalism by using only bounded functions without advanced Schwartz distributions. Nevertheless, the step-function expression has not been extended so far compared with the application of the delta-function expression. Here we demonstrate a unified formula that extends this step-function expression for single-variable functions to multiple-variable functions. It can be interpreted as the decomposition of any multivariate function with respect to the Heaviside step function.

## 2. Decomposition of multivariate functions using the Heaviside step function

### 2.1. Definition

Let *R*(*X*_1_, *X*_2_, …, *X*_*N*_) be a continuous real function defined for 0 ≤ *X*_*i*_ < ∞ and satisfies:

-  Whose derivatives  exist and continuous where *α* = *α*_1_ + ⋯ + *α*_*N*_ and *α*_*i*_ ≥ 0 is a natural number.
 
 can be integrated with respect to a given *X*_*i*_ while the other variables are held fixed.
- Sequences of functions  uniformly converge to  for *X*_*j*_ and *j* ≠ *i*.

### 2.2. Premise

We follow Dirac’s method:

-  The Dirac delta function is regarded as a function (not a Schwartz distribution)
-  The derivative of the Heaviside step function is regarded as the Dirac delta function

### 2.3. Theory

We demonstrate that the defined function *R*(*X*_1_, *X*_2_, ⋯, *X*_*N*_) can be decomposed into:
2.1

where


defines a set of Heaviside step functions for each *μ*_*i*_ ≥ 0; ; *α* = *α*_1_ + ⋯ + *α*_*N*_; and *α*_*i*_ = 0 *or* 1.

### 2.4. Proof

The formula (2.1) may be expressed as:
2.2

We use mathematical induction.

Using the definition of the Dirac delta-function δ, then for any real function *R*(*X*_1_, *X*_2_, ⋯, *X*_*N*_)
2.3

Therefore,
2.4

with *X*_*i*_ ≥0.

The expression (2.2) for single-variable functions (*N* =1),
2.5

holds by Lemma 2.1 given below. Note that, for single-variable functions, Dirac described (Dirac 1958) the essentially equivalent expression (1.2).

To initiate the mathematical induction procedure, suppose that the expression (2.2),
2.6

holds for some *N*. Using (2.6), the following,
2.7

holds because *R*(*X*_1_, ⋯, *X*_*N*_, *μ*_*N* + 1_) can be regarded as one of the *R*(*X*_1_, ⋯, *X*_*N*_) appending a parameter *μ*_*N* + 1_. Multiplying both sides of (2.7) by *δ*(*μ*_*N* + 1_ - *X*_*N* + 1_) and then integrating each term with respect to *μ*_*N* + 1_, one obtains on the left-hand side,
2.8

and on the right-hand side,
2.9

The order of the integrations can be changed because each integrand can be integrated with respect to its corresponding *μ*_*i*_ while holding other variables fixed. Therefore, (2.9) can be transformed into
2.10

The terms enclosed in braces in (2.10) can be transformed using Lemma 2.1 as follows,
2.11

where Lemma 2.2 was also used.

Finally (2.11) becomes
2.12

Thus, assuming expression (2.6) for *N* leads to the same expression for *N* +1. The formula for *N* =1 also holds as described above. Therefore, (2.1) holds for any natural number *N*.

**Figure 1:**
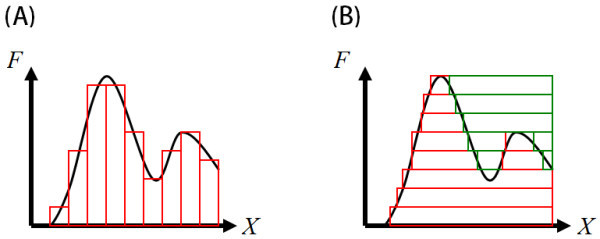
**Equality between (A) sums of vertical stripes and (B) sums of horizontal stripes (positive (red) and negative (green) contributions) under the target integrand.**

The key is transforming integrands with Dirac delta functions to ones with Heaviside step functions. It is represented by Lemma 2.1, which is intuitively understandable with equality between sums of vertical stripes and sums of horizontal stripes under the target integrand *F* (Figure 1).

### 2.5. Lemmas

#### Lemma 2.1

2.13

where *F* is *R* or its derivatives.

Proof.

From (A1.2),


If *X*_*i*_ >0,


With *σ*(*X*_*i*_ - ∞) = 0 and *σ*(*X*_*i*_ - 0) = 1, we obtain


If *X*_*i*_ =0, the left-hand side of (2.13) is


whereas the right-hand side of (2.13) is


(Q.E.D.)

#### Lemma 2.2



where *a* = *a*_1_ + … + *a*_*N*_ and *a*_*i*_ ≥ 0 is a natural number.

Proof.

Since derivatives of *R* is continuous,
2.14

Where (*r*_*h* → 0_) is a sequence of functions. The sequence of function (*r*_*h* → 0_) converges uniformly to  for *μ*_*j*_ and *j* ≠ *i*. Similarly  converges pointwise for *h* to  since it is continuous. Therefore, the order of the limits can be interchanged (Rudin 1976). Finally (2.14) be


(Q.E.D.)

## 3. Conclusions

We have demonstrated the decomposition of a multivariate function as a sum of integrals of which each integrand is composed of a derivative and a direct product of Heaviside step functions. The expression offers a rigorous formalism by using only bounded functions without the Dirac delta and Schwartz distributions; applications in approximation theory of functions using the Heaviside step or sigmoid functions with suitable parameters and dimensionality; and potent applications to mathematical methods in physics and engineering.

## Appendix

Some well-known formula related to the Heaviside step function appeared in the main text:
A1.1

Proof.

If *X* > 0,


If *X* < 0,


If *X* = 0,


(Q.E.D.)
A1.2

Proof.

From (A1.1),


Therefore, by differentiating both sides,


(Q.E.D.)
